# Simulated proximity enhances perceptual and physiological responses to emotional facial expressions

**DOI:** 10.1038/s41598-021-03587-z

**Published:** 2022-01-07

**Authors:** Olena V. Bogdanova, Volodymyr B. Bogdanov, Luke E. Miller, Fadila Hadj-Bouziane

**Affiliations:** 1 IMPACT Team, Lyon Neuroscience Research Center, INSERM, U1028, CNRS, UMR5292, University of Lyon, Bron Cedex, France; 2 Université de Bordeaux, Collège Science de la Sante, Institut Universitaire des Sciences de la Réadaptation, Handicap Activité Cognition Santé EA 4136, Bordeaux, France; 3 Donders Centre for Cognition of Radboud University in Nijmegen, Nijmegen, The Netherlands; 4 INCIA, CNRS UMR 5287, Université de Bordeaux, Bordeaux, France

**Keywords:** Neuroscience, Physiology, Psychology

## Abstract

Physical proximity is important in social interactions. Here, we assessed whether simulated physical proximity modulates the perceived intensity of facial emotional expressions and their associated physiological signatures during observation or imitation of these expressions. Forty-four healthy volunteers rated intensities of dynamic angry or happy facial expressions, presented at two simulated locations, proximal (0.5 m) and distant (3 m) from the participants. We tested whether simulated physical proximity affected the spontaneous (in the observation task) and voluntary (in the imitation task) physiological responses (activity of the corrugator supercilii face muscle and pupil diameter) as well as subsequent ratings of emotional intensity. Angry expressions provoked relative activation of the corrugator supercilii muscle and pupil dilation, whereas happy expressions induced a decrease in corrugator supercilii muscle activity. In proximal condition, these responses were enhanced during both observation and imitation of the facial expressions, and were accompanied by an increase in subsequent affective ratings. In addition, individual variations in condition related EMG activation during imitation of angry expressions predicted increase in subsequent emotional ratings. In sum, our results reveal novel insights about the impact of physical proximity in the perception of emotional expressions, with early proximity-induced enhancements of physiological responses followed by an increased intensity rating of facial emotional expressions.

## Introduction

Perception of the objects surrounding us is affected by their physical proximity^1^. Their spatial localization is based on the ability of the visual system to evaluate the distance between the observer and the object. In the case of a familiar object, both its perceived angular size and pre-existing knowledge of its size may help predict its spatial location^2^. Faces are among such objects and are often used in social neuroscience. In particular, facial expression recognition might be modulated by the size or perceived proximity of the face, despite some inconsistencies. For instance, in the study of Wallbott^3^ neither emotion recognition nor intensity rating was influenced by the size of the stimuli (full screen, 1/4, 1/9 and 1/16 size of the screen), presented at about 3 m from the observer. The emotions stay recognizable even at very long distances up to 100 m^4^. Other studies show that emotion recognition was impacted by stimulus size (simulated viewing distances ranged from 3.3 to 105.6 m) and by the image frequency spectrum^5^, or by the image resolution^6^, with smaller faces taking longer to categorize and to discriminate than larger ones^7,8^. Importantly to the current study, the perceived intensity of the emotional facial expression may also be affected by the distance of the face stimulus^9,10^, however the exact mechanisms underlying this modulation in relation to space is currently poorly understood.

The perception of facial emotions combines both conscious and unconscious components. Among unconscious responses, spontaneous facial mimicry is thought to be involved in embodied recognition of emotions^11,12^, i.e. understanding the emotional state of others through an activation of one own corresponding mental state^13,14^. For instance, EMG activity of face muscles changes when viewing static or dynamic pictures of facial emotions^15–17^*.* Such is the case of the activity of the *corrugator supercilii* muscle, the upper face muscle, when viewing angry face images. That EMG response is thought to be related to the emotional intensity rating scores participants attribute to the emotional stimuli^18^. Experimentally blocking facial muscles^19–21^ or pathological conditions^22–24^ that affect the muscles controlling facial expressions lead to a reduction of emotional recognition and emotional intensity ratings^11,12^. Congruent social information (positive or negative social labeling) improves facial mimicking responses, while incongruent social information decreases valence and arousal ratings^25^. Combined EEG–EMG study revealed that early visual processing of facial expressions may determine the magnitude of subsequent facial imitation^26^ and, thus, possibly impacts facial emotional evaluation.

In the present study, we sought to determine whether the perceived distance of facial emotion alters conscious and unconscious emotional processes and in particular the EMG facial responses. Stimuli situated within near, reachable, or socially relevant distances benefits from privileged perceptual processing^27,28^. This region of space near the body where physical interactions with objects in the environment take place, has been termed PeriPersonal Space (PPS)^29^ and is sensitive to social stimuli^30^. Recent evidence suggests that beyond perceptual effects, social stimuli within PPS elicit valence-dependent physiological changes^8,31,32^ which may impact emotional perception via interoceptive mechanisms^33^. In the same vein, approaching people induce a sense of discomfort that allows regulating interpersonal space distances between individuals^34^. In that context and whether referring to the close space surrounding the body in terms of PPS or a social interpersonal space, we hypothesized that the perception of proximal, compared to distal, emotional faces will intensify emotional embodiment, by simulating corresponding affective states in participants^13,35,36^. Our paradigm used a looming human-like shaped mask to cue a strong sense of intrusion into proximal space in the observer. We then displayed brief videos of faces of respective sizes displaying either anger or happiness and the participants were instructed to either observe (task 1) or imitate these emotional expressions (task 2). Dynamic facial expressions are rated as more intense^15,16^ and induce stronger response of face muscles compared to static ones^17^*.* We measured continuous *corrugator supercilii* activity and pupillary response, two physiological correlates of emotional perception^15,18,26,37^ during and after the presentation of dynamic emotional videos to obtain an objective information about the temporal profile of evoked responses. Sensitive to subjective arousal, pupillary response may reflect autonomic reactivity to emotions^37^. Finally, in order to probe how the subjective components of emotional perception varied as a function of the face size, we also measured the participants’ emotional intensity rating. We predicted that proximal compared to distant faces would enhance the perception of emotional intensity, reflected in physiological and behavioral measures, during both observation and voluntary imitation of emotional stimuli.

## Results

In two tasks, forty-four healthy participants were shown brief videos of angry or happy faces. The videos were presented in two conditions, proximal or distant, respectively simulating distances of 0.5 or 3 m from the participants. We studied how the condition of presentation of the emotional stimuli modulates several facets of emotional perception: *corrugator supercilii* face muscle activity, pupil size and emotional intensity ratings, the latter being provided at the end of each trial. In addition, in the second task, before the rating, subjects were asked to imitate the facial expression displayed on the screen. In both tasks, we found in proximal compared to distal condition, an enhanced response of the *supercilii* face muscle and a larger pupil size variation, followed by higher ratings of emotional intensity. The main results are summarized in Table 1 and are presented in more details below.

**Table 1:** Summary of the main results.

Experimental measures (*statistical tests)*	Tasks	
	Observation	Imitation
Amplitude of the Corrugator supercilii EMG activity *(Cluster-based permutation)*	Happy expressions: Significant decrease in EMG activity, more pronounced in proximal compared to distal conditions *(p* < *0.001)* Angry expressions: No significant changes in EMG activity	Happy expressions: Significant decrease in EMG activity, more pronounced in proximal compared to distal conditions *(p* < *0.05)* Angry expressions: Significant increase in EMG activity, more pronounced in proximal compared to distal conditions *(p* < *0.001)*
Latency of the corrugator supercilii EMG response to angry faces * *(Repeated measures ANOVA)*	N/A	Angry expressions: Faster latency for proximal compared to distal conditions *(p* < *10*^*–9*^*)*
Difference in pupil size for angry *versus* happy faces *(Cluster-based permutation)*	Angry *versus* Happy expressions: Larger pupillary affective response in proximal compared to distal conditions, in both the observation and imitation tasks (p < 0.05) No significant difference between tasks	
Emotional intensity ratings *(Repeated measures ANOVA)*	Main effect of condition *(F(2,40)* = *22.69; p* < *10*^*–5*^*)*: stronger rating in proximal compared to distal conditions Main effect of task *(F(2,40)* = *3.51; p* = *0.039)*: stronger rating in the imitation compared to the observation task No interaction between factor “condition” and factor “task”	

*For the imitation of the angry expressions, the signal-to-noise ratio in EMG activity allowed us reliably identify the onset latency of EMG responses in distant or proximal condition.

### Corrugator supercilii muscle activity

**Figure 1:**
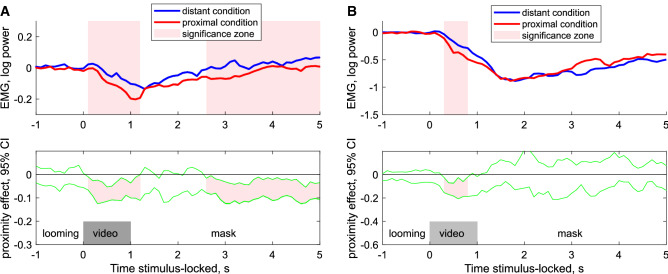
C*orrugator supercillii* activity during observation (**A**, n = 41) and imitation (**B**, n = 36) of happy faces. Upper row: the median of EMG activity for distant and proximal conditions. Lower row*:* 95% confidence intervals for effect of condition (proximal vs distant conditions). *Pale pink shadow* indicates clusters of data points with significant differences between proximal and distant conditions (A - p < 0.001 and B - p < 0.05 cluster level based on median bootstrap permutation test, n = 1000).

**Figure 2:**
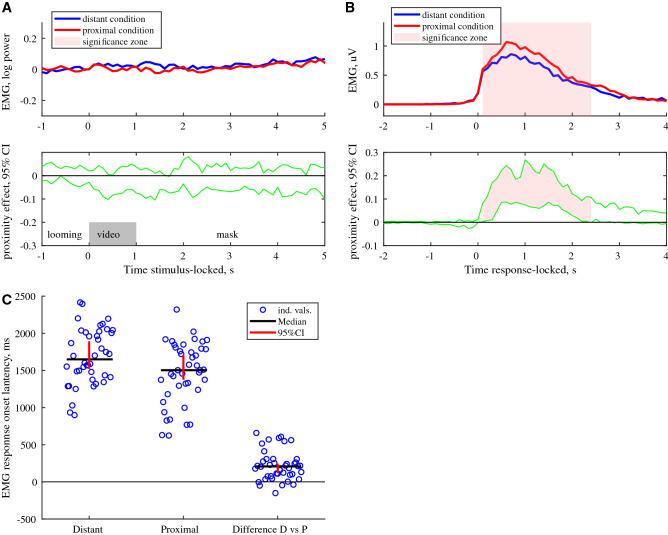
C*orrugator supercillii* activity during observation (**A**) and imitation of angry faces (**B**,**C**) (n = 41). (**A**) *Corrugator supercillii* activity during the observation of angry faces (n = 41); Upper row: the median of EMG activity for distant and proximal conditions. Lower row: 95% confidence intervals for effect of condition (proximal vs distant conditions). (**B**) *Corrugator supercillii* activity during the imitation of angry faces (n = 41), responses locked to onset of the start of imitation activity; Upper row: the median of EMG activity for distant and proximal conditions. Lower row*:* 95% confidence intervals for effect of condition (proximal vs distant conditions). *Pale pink shadow* indicates cluster of data points with significant differences between proximal and distant conditions (p < 0.001 cluster level based on median bootstrap permutation test, n = 1000). **(C)** Latency of the *corrugator supercillii imitation* response onset for the distant condition (small faces) or the proximal condition (large faces) and the effect of condition (distant vs proximal condition. Black lines indicate average latencies; red bars mark 95% CI.

During both tasks (observation and imitation), we measured *corrugator supercilii* muscle activity while participants were viewing dynamic emotional (angry or happy) face stimuli, to estimate the engagement of the muscle in unconscious and conscious emotional mimicking, correspondingly. We found that activity of the *corrugator supercilii* muscle differed depending on the emotional content of the video, condition, and task (Figs. 1, 2, Supplementary Fig. S2). During the observation task, happy faces evoked an early significant decrease of *corrugator supercilii* muscle activity which was long-lasting and more pronounced in the proximal compared to distal condition (p < 0.001 of cluster significance, Fig. 1A). Active imitation of happy emotional expressions also yielded a decrease of *corrugator supercilii* muscle activity in most of the subjects (n = 36), which was significantly greater for faces presented in proximal compared to distant trials (p < 0.05 of cluster significance, Fig. 1B).

Angry faces did not evoke any significant EMG response in the observation task (Fig. 2A), while imitation of dynamic angry emotional expressions caused strong activation of the *corrugator supercilii* muscle (Fig. 2B). Taking advantage of the large response amplitude during the imitation of angry faces, we were able to estimate the latency of the individual responses and to compute the latency-locked EMG responses (Fig. 2C). There was a larger amplitude of *corrugator supercilii* muscle activity even when potential contribution of the onset latency was eliminated (p < 0.001 of cluster significance). The latency of this response was faster for faces presented in the proximal than in distant condition (1467 ± 66 and 1690 ± 59 ms respectively) and this difference was highly significant (F = 52.0, p < 10^–9^, Fig. 2C).

**Figure 3:**
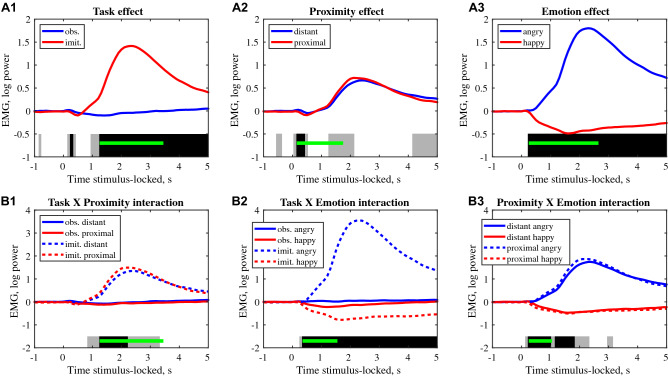
EMG activity, repeated measures ANOVA with three factors: Task (imitation and observation), Proximity condition (distant and proximal) and Emotion (angry and happy), two levels each (N = 41). Top raw (**A1**, **A2**, **A3**): The averaged EMG activity illustrating main effects: Task, Proximity and Emotion. Bottom raw (**B1**, **B2**, **B3**): The averaged EMG activity illustrating interaction effects: Task × Proximity, Task × Emotion and Proximity × Emotion. The contiguous temporal clusters of significant effects are shown with gray (p < 0.05) and black (p < 0.001) bars at the bottom of each plot. The cluster-level extent thresholds (computed by cluster permutation method, p < 0.001) are indicated green lines.

Significant main effect of emotion and its interaction with proximity is also confirmed with the three-factor repeated measures ANOVA (Fig. 3, A3, B3, p < 0.001 at the cluster level). The dynamics of the effects demonstrate early onset, 200–300 ms after the emotional video onset. This analysis also confirmed the main effect of task, with a high amplitude of EMG activity during the imitation of angry expressions (Fig. 3, A1), which clearly unfolds in a highly significant [Task × Emotion] interaction (Fig. 3, B2, p < 0.001 at the cluster level).

The average curves in the [Task × Emotion] interaction confirm that the most prominent increase in corrugator supercilii EMG amplitude was observed during the imitation of angry expressions and that the decrease of EMG activity was higher for imitation than for observation of happy expressions (Figs. 2, 3, B2).

### Pupillary affective response

**Figure 4:**
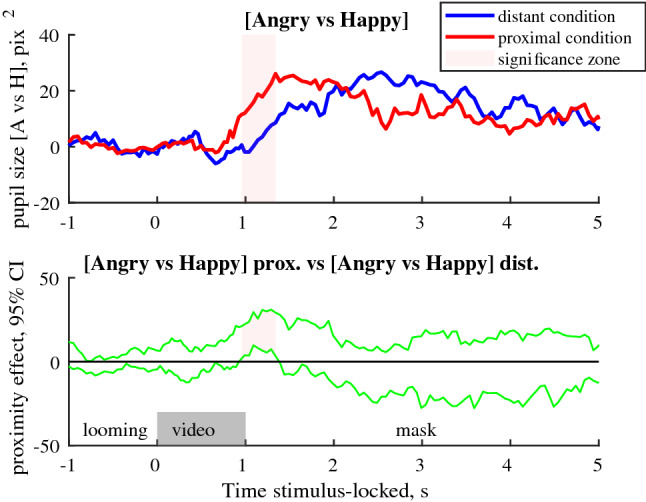
Upper row: Median differential pupillary affective response, estimated as a difference between pupil size changes in trials with angry and happy faces for distant or proximal conditions in both tasks (n = 42). Lower row: 95% confidence intervals for effect of condition (proximal vs distant conditions). Pale pink shadow indicates cluster of data points with significant differences between proximal and distant conditions (p < 0.05 cluster level based on median bootstrap permutation test, n = 1000).

Angry faces resulted in a larger pupil size than happy faces in both the observation and imitation tasks, and we found no significant difference between these tasks (Supplementary Fig. S3). The difference between pupil size in trials with angry versus happy faces, termed thereafter the ‘pupillary affective response’, was further modulated by the condition of presentation. In trials where faces were presented in proximal condition, in both the observation and imitation tasks, the pupillary affective response was larger during the first second after the offset of the emotional video (p < 0.05 of cluster significance, Fig. 4).

### Perceived intensity of emotional expressions

**Figure 5:**
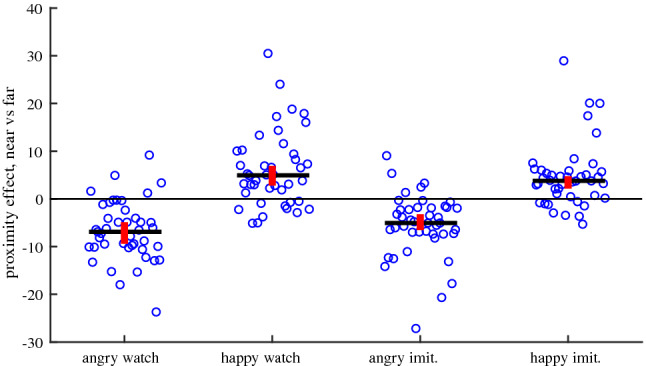
Effect of condition on emotional intensity ratings (difference between proximal and distant conditions) for happy and angry facial expressions, (n = 42, p < 10^–5^). Black lines indicate median proximity condition effect; red bars mark 95% CI.

The emotional intensity ratings for happy and angry facial expressions are presented in Table 2. A repeated measures ANOVA analysis revealed a significant effect of condition (F(2,40) = 22.69; p < 10^–5^) in emotional intensity ratings (ranged from 0 to 90 degrees in the scale, see the methods section and Fig. 7 for detailed description): 63.5 ± 1.7 for proximal condition and 57.6 ± 1.6 for distant condition. There was also an effect of the task (F(2,40) = 3.51; p = 0.039), with a slightly stronger emotional intensity rating during the imitation task (61.9 ± 1.8) than the observation task (59,2 ± 1.6); but no interaction between task and condition was found. In Fig. 5, the effect of proximity is presented as the difference between the two conditions (proximal versus distant) for both tasks.

**Table 2:** Emotional intensity ratings for happy and angry facial expressions (mean ± standard error of mean, n = 42).

	Distant condition	Proximal condition	Difference [Proximal vs Distant]
Angry observation	− 55.9 ± 1.5	− 62.5 ± 1.8	− 6.6 ± 1.0
Angry imitation	− 59.2 ± 1.8	− 64.5 ± 1.9	− 5.3 ± 1.0
Happy observation	58.3 ± 2.1	64.2 ± 2.1	5.9 ± 1.2
Happy imitation	59.6 ± 2.2	64.2 ± 2.2	4.6 ± 1.0

### Within-subject relationship between condition-dependent changes in emotional intensity rating and corrugator supercilli activation during imitation of angry expressions

Using the most significant and consistent proximity-driven effect, i.e. the increase of EMG activity of the *corrugator supercilii* muscle during the imitation of angry expressions, we aimed to investigate how this change may be related to condition-dependent changes in emotional intensity ratings.

**Figure 6:**
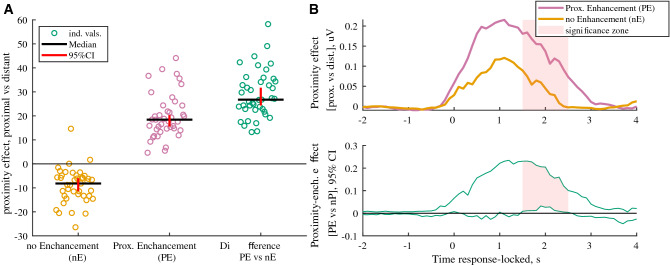
Within-subject relationships between the proximity effect on face muscular activity and emotional intensity ratings during imitation of angry facial expressions (n = 41). **(A)** The magnitude of condition-related differences in emotional intensity ratings (proximal vs distant condition) for angry identities estimated for two sub-categories of trials (proximity-enhancement, PE and no-enhancement, nE sub-category, respectively). The subject-wise average proximity effects shown for nE, PE trials and the difference [PE vs nE], as well as the group medians and 95% CI. **(B)** Upper row: Median differential (proximal vs distant) corrugator supercilii EMG activity, which is locked to the response onset for PE and nE trials. Lower row: the 95% CI of the difference [PE vs nE]. *Pale pink shadow* indicates cluster of data points with significant difference between PE and nE trials (p < 0.01 cluster level based on median bootstrap permutation test, n = 1000).

To do that, for each subject (n = 41), we estimated the upper and lower terciles of the proximity-induced differences in emotional intensity ratings for angry identities to dissociate trials according to the size of ‘proximity effect’: (1) those with proximity-induced effect above the upper tercile (PE, proximity enhancement trials) and (2) those with proximity-induced effect below the lower tercile (nE, no enhancement trials) (Fig. 6A). We then estimated the effect of proximity condition on EMG activity in those trials for each subject. It turned out that in PE trials, corresponding to the largest difference in ratings, there was a significantly greater EMG enhancement in proximal versus distant condition compared to nE trials (p < 0.01, Fig. 6B). This effect was the most significant at the second part of the EMG response i.e. 1.5–2.5 s after the onset of the response, suggesting prolonged muscle activation in PE trials.

## Discussion

The aim of the present study was to test whether the previously documented impact of proximal *versus* distant faces on emotional intensity rating^10^ was associated with early increased physiological responses both during observation and imitation of facial emotional expressions. We presented to healthy volunteers videos of angry or happy faces in conditions simulating proximal or distant locations. As previously shown^15^, observation and imitation of happy faces evoked a decrease in *corrugator supercilii* activity, while imitation of angry faces evoked an increase of EMG activity of that face muscle. Importantly, we provide here the first empirical evidence of an enhanced response of *corrugator supercilii* activity for faces presented in proximal compared to distant conditions while passively viewing or imitating the emotional videos. We also found changes related to the emotional content of the video in the pupillary size that were more pronounced for proximal as compared to distant conditions. These physiological signatures were followed by emotional intensity ratings that were also more marked in proximal as compared to distant conditions. As the engagement of the upper face muscles is essential for emotional perception^11,19,38,39^, and disruption of facial mimicry selectively impairs recognition of emotions^12,20^, we postulate that proximity-related increase in the activity of the face muscles while viewing or imitating facial emotional expressions facilitates emotional embodiment, that lead to enhanced perception of emotional intensity.

It is known that the *corrugator supercili* muscle response to facial emotional expressions is related to empathy, the ability to understand others’ emotions^40^. People with high empathic traits are more accurate in imitating facial expressions^41^ and exhibit a higher level of autonomic arousal during emotional stimulation^42^. However, the degree of empathic responses depends on the spatial distance from another person^43^. The stronger response of the *corrugator supercili* muscle in proximal compared to distant conditions during both passive viewing and imitation of the emotional videos in our results suggests that proximity may enhance interpersonal emotional resonance, i.e. experiencing a similar emotional state^44^ and thus impact empathic response.

In our study, we specifically probed voluntary imitation. The imitation skill emerges in early infancy^45^ and play a key role in social interactions which are deficient in a number of neurological disorders^46^. We demonstrated that activation of the *corrugator supercilii* muscle during imitation of angry expression was both stronger and faster for proximal than distant condition. This rapid EMG response may be related to the fast visual processing in PeriPersonal Space network (PPS), that mainly relies on the dorsal visual stream, optimized for action^1^. In support of that, proximal emotional faces are scanned with a different eye movement strategy^9^. Peculiarities in visual processing of facial expressions may determine the magnitude of subsequent facial muscle response^26^, that in turn could affect emotional perception^36^. Therefore, emotional perception of proximal stimuli may benefit from PPS-related facilitation, which helps to react to slight and rapid changes in facial expressions of people nearby. The rapid response of face muscles might reflect a covertly set imitation program which is executed as soon as the emotion is recognized by the brain, and this program incorporates the proximity information. Such an interpretation is supported by the ability to mimic masked emotional expressions, which are not consciously perceived^47^. It is therefore possible that faster and more efficient imitation of emotional facial expressions presented in proximity (vs. at distance) may facilitate social interactions. We found that EMG response lasted much longer than the stimulus presentation, thus it might also be related to the maintaining of the emotional impression in memory^48^. We also found that a greater engagement of the *corrugator supercilii* in the imitation of angry expression predicted greater changes in emotional intensity ratings. Interestingly, for trials with greater behavioral proximity effect, the EMG response was larger and lasted longer. This is in line with several previous findings demonstrating a relationship between EMG activity of *corrugator supercilii* and subjective ratings of dynamic facial emotional stimuli^18,49–51^**.** One needs to mention that a repeated exposure to the same stimuli may be a confound when evaluating emotional intensity. In the current study, we were interested in the effect of proximity and not specifically in the effect of the task (observation vs imitation) that may be a promising direction for further research. Another potential future avenue is to sort out potential confounds in activation of the *corrugator supercilii* due to varied difficulty of the emotional judgement task as a function of proximity.

The proximity-related increase in EMG activity was accompanied with an enhancement of pupillary affective responses, preceding the emotional intensity ratings. The pupillary affective response was estimated as a difference between the pupil size in trials with angry and happy expressions, separately for proximal and distant condition, thus, minimizing effects of low-level image properties. We found that proximity increased pupillary affective response and this may suggest an increased difference in arousal evoked by angry and happy expressions that depends on the perceived distance^31,52^. This finding is in line with other changes in autonomic activity induced by the size of emotional stimulus^53^ and valence-dependent physiological changes induced by the stimuli within the close space^8,31,32,54^. The difference between two conditions was significant during the first second after the emotional video, likely reflecting a fast, automatic response of the autonomic nervous system.

Why do we perceive proximal emotional faces as more intense? The interpretation of our and earlier findings^9,10^ could not be explained only by differences in early visual processing^8^. Since closer faces occupy larger retinal size, they can induce more arousal and greater concentration of attention^31,55^. However, it has recently been shown that stimuli discrimination is facilitated in PPS, even after retinal size correction^8,56^. This finding points towards the importance of other depth cues for the perception of stimuli in near space. We introduced a four second delay between the presentation of emotional stimuli and the rating of the emotional intensity. This allowed a relatively greater impact of top-down processes in the following perceptual judgement. In spite of a very significant effect at the group level, a subset of subjects did not provide any increased ratings for faces presented in proximal conditions (Fig. 5), which also argues that the size of the stimuli, alone, does not firmly determine the judgment about the intensity of emotional expression.

**Figure 7:**
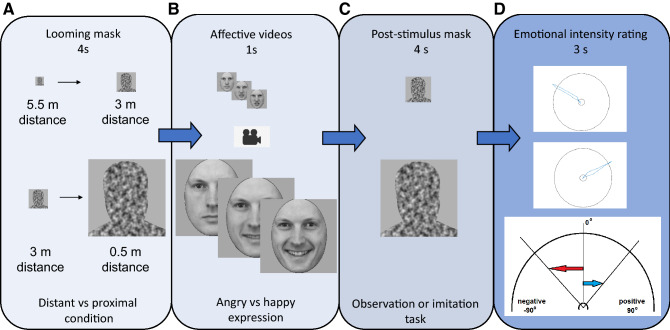
Experimental trial description. (**A**) Four seconds of a gradually expanded head and shoulders noisy silhouette mask with a constant velocity of slow walking speed around 0.6 m/s. The human-like mask silhouette was chosen to induce the illusion of a person approaching towards the observer. (**B**) One second of affective video clips either of 4° size (distant condition) or 26° size (proximal condition). For the second session, participants were asked to imitate observed expression as soon as they see it. (**C**) Four seconds of post-stimulus mask whose size was equal to the previous video. (**D**) Three seconds of emotional judgment task. See detailed descriptions in the text.

In general, the concept of proximity in its social meaning reflects trust and attachment to the partner or perceived psychological distance between self and others^57^. Not surprisingly, people typically prefer to keep larger distances with strangers and closer distances with friends or family members^58^. Social proximity may be related to greater self-other overlap, that facilitates imitation^59^ and correlates with response in near space^60^. What are the mechanisms underlying such effects? It is possible that a framework, integrating proximity-dependent spontaneous and voluntary responses underlies such effects. In other words, it is possible that in our proximal condition, the physical proximity may evoke a sense of social closeness and this increased emotional perception, potentiated imitation response and augmented emotional judgements. Our experimental design (Fig. 7) used features of the stop-distance paradigm, where an approaching stranger intrudes into near space, evoking defensive-like reaction reflecting itself in a sense of discomfort^61^ and modulates physiological indexes^54^. We cannot disentangle the mere effect of the physical proximity from the effect of an intrusion into the personal space which may trigger anxiety and a sense of discomfort^61^, but it can be stated that overall physical and social proximity may yield an increase in physiological and behavioral responses^32,54^*.*

Previous studies have shown that large or approaching faces evoke activation of fronto-parietal areas^62,63^ which are part of the brain’s PPS network^64^. Its activation facilitates interactive and defensive responses to stimuli in near space^27,65^ and can be modulated by social factors^8,60^. It is thus possible that proximal condition used in our study triggers a greater activation of the PPS network compared to distant condition; thus, behavioral and physiological changes observed here might reflect a signature of this increased PPS activation. One of the neuronal hubs which might further come into play in the interaction between space and emotion is the amygdala. Several recent studies have demonstrated a tight coupling between the activity of the amygdala and that of the fronto-parietal PPS network during the perception of social stimuli: emotional sounds that trigger amygdala activation^66^ also modulate responses of the PPS network^65^. Theta-burst stimulation of the PPS brain areas (ventral premotor cortex and inferior parietal lobule), boosts amygdala’s response to emotional stimuli^67^. Noteworthy, the amygdala is essential to regulate social distances^68^ and not surprisingly its activity is enhanced with looming stimuli invading this space^69^. The activation of the amygdala correlates with activity in the *corrugator supercilii* muscle during observation of emotional expression^70^; the presence of proprioceptive feedback from the face muscles seems to be a necessary condition for amygdala activation in response to emotional stimuli^71,72^. Taken together, it is tempting to suggest that the enhanced emotional and physiological responses to proximal faces found in the present study might be subserved by the functional interplay between the PPS network and the amygdala, a hypothesis that might be tested in future neuroimaging studies.

## Conclusion

The present study explored the effects of proximity in emotional perception and provided some insights into the dynamic of such response. We propose that proximity-driven increase in automated and voluntary face muscle activity could amplify emotional perception and participate in adequate responses to the social presence in proximity during social interactions. A greater differential affective pupillary response to proximal faces confirms greater arousal variation related of such proximal encounters. Together, these results emphasize the role of proximity as an important factor for the regulation of social behaviors likely governed by reciprocal interactions between affective and spatial perception networks, which may be of particular interest in light of the growing usage of telecommunication and video-conferencing in the modern world^73^.

## Methods

### Participants

Forty-four healthy volunteers (18 men, 26 women, M = age: 26.4, SD = 5.1 y.o) participated in the study. Based on the EMG study of Kunecke et al., 2014^18^ the critical sample size for our study was 44 subjects as computed with G*Power 3^74^ for the effect size (f) 0.36, power of 0.90, p < 0.05 and two measures in repeated measures ANOVA, assuming no overall correlation between the measures. Based on the pupillometry study of Burley et al., 2017^37^ the critical sample size for our study was 44 subjects for the effect size (f) 0.18, power of 0.90, p < 0.05 and six measures in repeated measures ANOVA, assuming correlation between the measures of 0.5. All subjects provided written informed consent. None of the them had a history of neurological or psychiatric disorders, and the vision of all subjects was normal or corrected-to-normal. Two of the participants did not correctly follow the instructions provided for the judgment task; their results were therefore excluded from all the analysis. The study followed the Declaration of Helsinki standards and was approved by the Institute National de la Santé et de la Recherche Médicale (INSERM) Institutional Review Board (IRB 00003888).

### Apparatus and experimental setup

A DELL 2208WFP 22’ monitor (resolution 1680 × 1050) was installed at 50 cm from the participant’s eyes. The subject’s chin was placed on a chinrest to align subject’s eyes with the center of the monitor. The activity of the *corrugator supercilii* muscle was recorded using the Bagnoli-2 EMG system (DELSYS). The bipolar electrodes were attached with the electrode-specific stickers (DELSYS) over the right *corrugator supercilii* muscle of the face^75^. The ground electrode was attached in the center of the forehead. The EMG signal was amplified (X10k), filtered (20 to 450 Hz), and digitized with a Power 1401 MK II data acquisition system and Spike2 software (Cambridge Electronic Design Limited) at a sampling rate of 1000 Hz. An EyeLink Portable Duo (SR Research) was used to monitor pupil size at a sampling rate of 20 Hz, which is sufficient to detect slow pupil changes provoked by emotional arousal^52^. The experimental paradigm was programmed in Matlab R2016a (The MathWorks Inc.), using the Psychophysics Toolbox-3 extension^76^.

### Procedure

The experiments consisted of two sessions that only differed in the task given to the subjects. For the first, observation task, subjects were instructed to look attentively at the screen, observe the presented emotional expression, and then rate the intensity of the facial emotional expression presented in the video by moving the computer mouse towards the circular visual scale, crossing it and to bring it back in the center for the screen (Fig. 7D). The vertical axis of emotional intensity rating scale represents division between two different valences: positive and negative in demi-circular continuous scale of valence and emotional intensity, where − 90° (on X axis) represents the most intense rating in negative emotion, 0° (on Y axis) represents a neutral emotion and + 90°, the most intense rating in positive emotion. For the second, imitation task, subjects were asked also to imitate the perceived emotional expression as soon as the emotional expression became clear enough and then rate the intensity of the emotional expression presented as in the observation condition. The tasks were not counterbalanced to avoid effect of the familiarity on automatic mimicry, and therefore the experiment always started with the observation task.

We used one-second video clips of 20 different male identities from the Emotional Faces database (KDEF-dyn)^77^. On the video clips, the facial expressions changed from neutral to negative (anger) or positive (happy) emotions. Those emotional expressions are known to modulate the *corrugator supercilii facial muscle’s* activity^26^*.* All video clips were black and white, cropped to oval form (nose at the center), and equalized for total luminance with the gray background and between all the individual video clips.

The stimuli were positioned in such way that the eyes of the emotional faces were on the same level as the observer’s eyes. The stimuli were presented in two conditions: ‘distant’ (with small stimuli) and ‘proximal’ (with large stimuli). Each identity was presented four times during each session: two emotions (happy and angry) × two conditions (proximal and distant). The emotions and conditions were pseudo-randomized, such that each emotion (happy or angry) or condition (proximal or distant) was not presented more than three times in a row and each of the four possible combinations of condition and emotion was not presented more than to two times.

One experimental session lasted about 16 min (80 trials). Each trial was 12 s long and grouped in four blocks of four min each. Between the blocks, the subject was able to rest and resume with the session at their own pace.

#### Trial structure

Each trial consisted of four consecutive parts (Fig. 7)*:* Four seconds of looming mask, one second of stimulus presentation, four seconds of stable post-stimulus mask, and three seconds for the emotional judgement task.

##### Looming mask

Each trial started with a gradually expanding silhouette of a human head and shoulders. This created an impression of looming with a constant velocity of a slow-paced walking speed (0.6 m/s). The human-like looming silhouette was chosen to provide the impression of a person approaching the observer. Depending on the condition (distant or proximal), the starting and ending angular size of the mask varied. For the proximal condition, the initial angular size of the height of the mask was 4° and the final angular size was 26°, corresponding to a movement from 3 to 0.5 m perceived distance with a face of 23 cm in height. For the distant condition, the initial height of the mask was 2° and its final size was 4°, corresponding to movement from 5.5 to 3 m perceived distance with a face of 23 cm in height.

##### Video presentation

The masked stimulus was then replaced with a one-second clip depicting a person expressing one-of-two emotions (angry or happy). The size of the video corresponded to the final angular size of the mask presented before.

##### Post-stimulus mask

The human-like silhouette of the same size replaced the video for next four seconds.

##### Evaluation scale and emotional intensity rating task

At the end of each trial, a circular visual analogue scale was shown for three seconds. To rate the emotional expression, the subjects were asked to move a mouse from the initial point in the center of a circle towards its edge and to come back to the initial position. The range of the emotional intensity rating scale was assigned to an upper demi-circle contour with the range for negative expressions from 0° to − 90° (from least-to-most negative) and for positive expressions from 0° to 90° (from least-to-most positive). A neutral rating corresponded to 0° (vertically at the top of the circle).

Altogether, the approaching mask, dynamic emotional stimuli, and the emotional intensity rating task aimed to produce a strong impression of intrusion into the near space; this was verbally confirmed by our subjects after the experiment. Our experimental design intentionally implicated some features of the ‘stop distance paradigm’ where participants are asked to indicate their level of discomfort induced by an approaching intruder^61^. In the present study, the experimental measure we used was not a level of discomfort but instead the rating in terms of emotional intensity provided by the facial emotional stimuli to measure the impact of proximal stimuli on their subjective emotional perception depending on the condition (distant or proximal).

### Data analysis

We tested the effect of condition (proximal and distant) in the following variables. (1) *Corrugator supercilii activity* was assessed for the observation and imitation tasks as a function of time and, in addition, for imitation of angry facial expressions, the latency of the imitation response was estimated. (2) The *affective pupillary response*, evoked by the presentation of emotional videos, was measured as the difference between angry and happy faces presented in the same condition, proximal or distant. (3) The rating of the *intensity of perceived emotional expressions.*

#### Corrugator supercilii (CS) activity

One subject did not follow the instructions in the imitation task and his EMG data were excluded from the analysis of the imitation activity. The preprocessing of the EMG signal was conducted following canonical practical recommendations^75^. In all conditions, except the imitation of angry expressions, the amplitudes of EMG activity were moderate, and were processed using the following processing pipeline^49^. We first estimated the EMG spectrogram using short-time Fourier transform. The Fourier-transform segment length was 100 ms with overlapping adjoining segments of 50 ms. Then, for each segment, the sum of the natural logarithms of the power spectral densities was computed for the frequencies 10 – 200 Hz, excluding the notch of 45–55 Hz. The archived time series was interpolated to the initial sampling rate (1 kHz) with shape-preserving piecewise cubic interpolation and then smoothed with 300 ms moving average. Eighty epochs were extracted (i.e. corresponding to 80 trials), each spanning from 4 s prior to the onset of the stimulus till 4 s after its offset for each task. Baseline correction was applied by subtraction of the average of the 4-s segment before the stimulus onset. Noisy epochs were identified by the minimum to maximum range at the 4 s baseline segment, and eight noisiest epochs were excluded for each subject.

##### Observing and mimicking happy faces

Among 41 subjects mimicking happy faces, we grouped in further analysis of 36 subjects, who demonstrated an overall decrease in averaged EMG activity during stimuli presentation and following four-second fixation compared to averaged activity during four-second baseline. The rest of the subjects (n = 5) demonstrated an opposite profile of CS response: an activation of the CS during the imitation of the happy facial expression was observed. In this subset of the subjects, we also found the effect of the proximity, which was reflected in a trend for greater activation of the muscle in proximal condition (Supplementary Fig. S1 in Supplementary materials). The average time-series for the two conditions (happy/distant or happy/proximal) was calculated for each subject in each task. Epochs were 100 times downsampled and the proximity effect estimated as a difference between the proximal and the distant conditions for happy stimuli in both observation and imitation tasks.

##### Observing and mimicking angry faces

The observation of angry expressions did not evoke any noticeable increase of EMG activity in both conditions (distant and proximal) (Fig. 2A). By contrast, for angry expressions in the imitation task, the amplitude of EMG activation of *the corrugator supercilii* allowed us to identify the onset of the muscle’s response in each trial, therefore we applied a different preprocessing algorithm. The recorded time series were first rectified, then second order local peak detected, interpolated at an initial sampling rate (1 kHz). We then performed epoching (− 4 to + 10 s respectively to the video onset) and baseline correction as described above. We estimated the maximum of the EMG anger imitation response for each trial of angry condition. Then we identified samples higher than the threshold of 20% of the average maximum amplitude of the anger imitation response.

Time point of the onset of the longest contiguous cluster exceeding this threshold is considered as the *latency* of the imitation response for each trial. This latency was averaged for each subject for proximal and distant condition and the difference between those values was computed for the proximity effect. A median-based bootstrap 95% confidence interval was estimated for the three measures in Matlab as well as repeated measures ANOVA analysis. The unsmoothed interpolated peak-detected and baseline-corrected epochs of the EMG activity were truncated as time-locked to the onsets of responses (two seconds before the response onset and four seconds after). As for happy expressions, the average time-series for the two angry conditions (angry/distant or angry/proximal) was calculated for each subject. Epochs were and 100 times downsampled and the proximity effect estimated as a difference between the reaction during imitation of proximal and the distant conditions.

#### Affective pupillary response

The algorithm of the preprocessing of pupil size data and automatic artifact rejection followed recommendations^78^. The upper threshold for artifact detection was set at 20 × median of the instantaneous changes in pupil size. All data points corresponding to instantaneous changes greater than this threshold were considered artifacts related to eye-blink onsets/offsets. All data-points with a pupil size less than 50 pixels were also regarded as blink-related artifacts. Each data point identified as an artifact (around 15% of samples in the window of interest from 1 s before to 5 s after of video presentation) and the two surrounding points were excluded from the analysis and replaced with spline-interpolated samples. In average 15% of the samples were rejected per epoch of 6 s.

80 epochs of 6 s were extracted starting one second prior to the onset of the video. The epochs were grouped into four conditions: angry/distant, happy/distant, angry/proximal, happy/proximal.

For each individual, out of the four conditions, we identified the two noisiest trials as those with the greatest within-trial minimum-to-maximum amplitude and excluded them from the subsequent analysis. The median time-series for each condition were calculated. Then, the emotion effect (here and above termed ‘affective pupillary response’) was calculated as the difference between angry and happy emotions, separately for distant and proximal conditions. The baseline correction was applied by subtracting the averaged 1 s pre-video baseline. The proximity effect was estimated as the difference between the proximal and the distant affective pupillary response.

#### Cluster level statistical analysis of the time series

A median-based bootstrap 95% confidence interval was estimated for each time point of the proximity effect in observation or imitation of happy expressions, imitation of angry expressions and differential affective pupillary response.

Cluster permutation method was used to correct for multiple comparisons in analysis of time series (1000 permutations)^79,80^. In order to correct for multiple non-independent measures, the temporally contiguous clusters of significant proximity effect in pupil size and EMG statistics were tested for the cluster level significance. We ran 1000 iterations as follows. (1) Random shuffling of proximal and distant condition labels of individual average time-series. (2) Estimation of the proximity effect as a difference between individual averaged proximal and distant time-series. (3) Calculation of the median-based group level 95% BCa bootstrap confidence interval for each data point of the proximity effect. The cluster volume p < 0.05, p < 0.01 and p < 0.001 thresholds was estimated as a 95^th^ percentile of the distribution of the maximal cluster volumes (sums of the distance between the 95% confidence interval edge and zero) of the temporally-contiguous clusters with condition assignment by chance.

##### Three-factor ANOVA of EMG activity

We conducted supplementary analysis using a classical three factor (task × proximity × emotion) repeated measures ANOVA in Matlab. The EMG signal was pretreated in a uniform way in forty-one subjects for all the conditions and the three-factor ANOVA was run for each time-point from − 1 s to + 4 s respectively to the stimulation onset. We tested for main effects of the factors Task (observation vs imitation), Proximity condition (proximal vs distant), Emotion (Angry vs Happy) as well as interactions [Task × Proximity], [Task × Emotion] and [Proximity × Emotion]. For the follow-up tests, we also used cluster-based permutation method and estimated for all significant (p < 0.001) clusters temporal extent with p < 0.001 threshold.

#### Rating of perceived intensity of emotional expressions

All subjects, except two, accurately recognized the emotional valence of the face stimuli regardless of their size. These subjects were excluded from further analysis. In the other subjects, misidentifications were rare, in average less than 2% of the trials and less than 10%. The trials with misidentifications were discarded from the analysis. The coordinates of the mouse cursor were continuously sampled (20 Hz) during the 3 s interval designated for the rating task. To estimate emotional intensity rating for a given trial, we first computed the maximal distance of the mouse cursor from the central starting position. The signed angular declination of this position relative to the center was taken as a measure of the perceived emotional intensity.

We used repeated measures ANOVA analysis in STATISTICA 13 (TIBCO) to estimate effects of the following factors of interest: (1) Task, (2) Condition. In addition, to estimate the effect of proximity, we first calculated the differences between the proximal and distant presentation and, finally computed the median-based group-level 95% confidence interval.

#### Relationships between proximity-driven changes in activity of the corrugator supercilii and intensity of perceived emotional expression in angry imitation trials

For each subject, we estimated the upper and lower tercile of the condition-induced differences in emotional intensity ratings. For each subject (n = 41) we defined two sub-categories of the trials: with proximity-induced differences greater the upper tercile for “proximity enhancement” (PE) or less important than the lower tercile, “no enhancement” (nE) trials. Then, in these sub-categories of trials, we estimated the median values of the EMG activity for the distal and proximal conditions, and the proximity effects [proximal vs distant condition] separately for the two sub-categories. The significance of the difference in proximity effects between two behaviorally defined sub-categories of trials was estimated with 95% median-based confidence interval and the cluster level statistics for time series. This analysis yielded significant differences between PE and nE trials only for angry expression imitation; other cases presented in Supplementary Fig. S4.

## Supplementary Information

Supplementary Information.

## References

[1] Blini E, Farnè A, Brozzoli C, Hadj-Bouziane F (2021). Close is better: Visual perception in peripersonal space. The World at Our Fingertips.

[2] Hosking SG, Crassini B (2010). The effects of familiar size and object trajectories on time-to-contact judgements. Exp. Brain Res..

[3] Wallbott HG (1991). The robustness of communication of emotion via facial expression: Emotion recognition from photographs with deteriorated pictorial quality. Eur. J. Soc. Psychol..

[4] Hager JC, Ekman P (1979). Long-distance of transmission of facial affect signals. Ethol. Sociobiol..

[5] Smith FW, Schyns PG (2009). Smile through your fear and sadness: Transmitting and identifying facial expression signals over a range of viewing distances. Psychol. Sci..

[6] Du S, Martinez AM (2011). The resolution of facial expressions of emotion. J. Vis..

[7] Cutting JE, Armstrong KL (2016). Facial expression, size, and clutter: Inferences from movie structure to emotion judgments and back. Atten. Percept. Psychophys..

[8] Dureux A (2021). Close facial emotions enhance physiological responses and facilitate perceptual discrimination. Cortex.

[9] Wang S (2018). Face size biases emotion judgment through eye movement. Sci. Rep..

[10] Gerhardsson A, Högman L, Fischer H (2015). Viewing distance matter to perceived intensity of facial expressions. Front. Psychol..

[11] Neal DT, Chartrand TL (2011). Embodied emotion perception: Amplifying and dampening facial feedback modulates emotion perception accuracy. Soc. Psychol. Pers. Sci..

[12] Borgomaneri S, Bolloni C, Sessa P, Avenanti A (2020). Blocking facial mimicry affects recognition of facial and body expressions. PLoS ONE.

[13] Prochazkova E, Kret ME (2017). Connecting minds and sharing emotions through mimicry: A neurocognitive model of emotional contagion. Neurosci. Biobehav. Rev..

[14] Wood A, Rychlowska M, Korb S, Niedenthal P (2016). Fashioning the face: Sensorimotor simulation contributes to facial expression recognition. Trends Cogn. Sci..

[15] Rymarczyk K, Biele C, Grabowska A, Majczynski H (2011). EMG activity in response to static and dynamic facial expressions. Int. J. Psychophysiol..

[16] Biele C, Grabowska A (2006). Sex differences in perception of emotion intensity in dynamic and static facial expressions. Exp. Brain Res..

[17] Sato W, Fujimura T, Suzuki N (2008). Enhanced facial EMG activity in response to dynamic facial expressions. Int. J. Psychophysiol..

[18] Künecke J, Hildebrandt A, Recio G, Sommer W, Wilhelm O (2014). Facial EMG responses to emotional expressions are related to emotion perception ability. PLoS ONE.

[19] Baumeister J-C, Papa G, Foroni F (2016). Deeper than skin deep: The effect of botulinum toxin-A on emotion processing. Toxicon.

[20] Oberman LM, Winkielman P, Ramachandran VS (2007). Face to face: Blocking facial mimicry can selectively impair recognition of emotional expressions. Soc. Neurosci..

[21] Wood A, Lupyan G, Sherrin S, Niedenthal P (2016). Altering sensorimotor feedback disrupts visual discrimination of facial expressions. Psychon Bull. Rev..

[22] Livingstone SR, Vezer E, McGarry LM, Lang AE, Russo FA (2016). Deficits in the mimicry of facial expressions in Parkinson’s Disease. Front. Psychol..

[23] Storbeck F, Schlegelmilch K, Streitberger K-J, Sommer W, Ploner CJ (2019). Delayed recognition of emotional facial expressions in Bell’s palsy. Cortex.

[24] Kordsachia CC, Labuschagne I, Andrews SC, Stout JC (2018). Diminished facial EMG responses to disgusting scenes and happy and fearful faces in Huntington’s disease. Cortex.

[25] Mermillod M (2017). Evidence of rapid modulation by social information of subjective, physiological, and neural responses to emotional expressions. Front. Behav. Neurosci..

[26] Achaibou A, Pourtois G, Schwartz S, Vuilleumier P (2008). Simultaneous recording of EEG and facial muscle reactions during spontaneous emotional mimicry. Neuropsychologia.

[27] Sambo CF, Iannetti GD (2013). Better safe than sorry? The safety margin surrounding the body is increased by anxiety. J. Neurosci..

[28] Canzoneri E, Magosso E, Serino A (2012). Dynamic sounds capture the boundaries of peripersonal space representation in humans. PLoS ONE.

[29] Rizzolatti G, Scandolara C, Matelli M, Gentilucci M (1981). Afferent properties of periarcuate neurons in macaque monkeys I. Somatosensory responses. Behav. Brain Res..

[30] Bogdanova OV, Bogdanov VB, Dureux A, Farnè A, Hadj-Bouziane F (2021). The peripersonal space in a social world. Cortex.

[31] Cartaud A, Ruggiero G, Ott L, Iachini T, Coello Y (2018). Physiological response to facial expressions in peripersonal space determines interpersonal distance in a social interaction context. Front. Psychol..

[32] Ruggiero G, Rapuano M, Cartaud A, Coello Y, Iachini T (2021). Defensive functions provoke similar psychophysiological reactions in reaching and comfort spaces. Sci. Rep..

[33] Critchley HD, Garfinkel SN (2017). Interoception and emotion. Curr. Opin. Psychol..

[34] Hayduk, L. A. PsycNET Record Display—PsycNET. *Personal Space: Where We Now Stand.* http://psycnet.apa.org/record/1984-01314-001?doi=1 (1983).

[35] Pinilla A, Tamayo RM, Neira J (2020). How do induced affective states bias emotional contagion to faces? A three-dimensional model. Front. Psychol..

[36] Lobmaier JS, Fischer MH (2015). Facial feedback affects perceived intensity but not quality of emotional expressions. Brain Sci..

[37] Burley DT, Gray NS, Snowden RJ (2017). As far as the eye can see: Relationship between psychopathic traits and pupil response to affective stimuli. PLoS ONE.

[38] Bulnes LC, Mariën P, Vandekerckhove M, Cleeremans A (2019). The effects of Botulinum toxin on the detection of gradual changes in facial emotion. Sci. Rep..

[39] Argaud S (2016). Does facial amimia impact the recognition of facial emotions? An EMG study in Parkinson’s disease. PLoS ONE.

[40] Harrison NA, Morgan R, Critchley HD (2010). From facial mimicry to emotional empathy: a role for norepinephrine?. Soc. Neurosci..

[41] Williams JHG, Nicolson ATA, Clephan KJ, de Grauw H, Perrett DI (2013). A novel method testing the ability to imitate composite emotional expressions reveals an association with empathy. PLoS ONE.

[42] Bogdanov VB (2013). Alexithymia and empathy predict changes in autonomic arousal during affective stimulation. Cogn. Behav. Neurol..

[43] Lomoriello, A. S., Meconi, F., Rinaldi, I. & Sessa, P. *Out of Sight out of Mind: Perceived Physical Distance Between the Observer and Someone in Pain Shapes Observer’s Neural Empathic Reactions*. arXiv:1808.01805 *[q-bio]* (2018). 10.3389/fpsyg.2018.01824 PMC6193079 30364280

[44] Robnett B (2004). Emotional resonance, social location, and strategic framing. Sociol. Focus.

[45] Jones SS (2009). The development of imitation in infancy. Philos. Trans. R. Soc. Lond. B..

[46] Gola KA (2017). A neural network underlying intentional emotional facial expression in neurodegenerative disease. Neuroimage Clin..

[47] Kaiser J, Davey GCL, Parkhouse T, Meeres J, Scott RB (2016). Emotional facial activation induced by unconsciously perceived dynamic facial expressions. Int. J. Psychophysiol..

[48] Pawling R, Kirkham AJ, Hayes AE, Tipper SP (2017). Incidental retrieval of prior emotion mimicry. Exp. Brain Res..

[49] Golland Y, Hakim A, Aloni T, Schaefer S, Levit-Binnun N (2018). Affect dynamics of facial EMG during continuous emotional experiences. Biol. Psychol..

[50] Tan J-W (2012). Repeatability of facial electromyography (EMG) activity over corrugator supercilii and zygomaticus major on differentiating various emotions. J. Ambient Intell. Hum. Comput..

[51] Sato W, Fujimura T, Kochiyama T, Suzuki N (2013). Relationships among facial mimicry, emotional experience, and emotion recognition. PLoS ONE.

[52] Bradley MM, Miccoli L, Escrig MA, Lang PJ (2008). The pupil as a measure of emotional arousal and autonomic activation. Psychophysiology.

[53] Codispoti M, De Cesarei A (2007). Arousal and attention: Picture size and emotional reactions. Psychophysiology.

[54] Candini M, Battaglia S, Benassi M, di Pellegrino G, Frassinetti F (2021). The physiological correlates of interpersonal space. Sci. Rep..

[55] Mishra MV, Srinivasan N (2017). Exogenous attention intensifies perceived emotion expressions. Neurosci. Conscious.

[56] Blini E (2018). Mind the depth: Visual perception of shapes is better in peripersonal space. Psychol. Sci..

[57] Beckes L, Coan JA (2011). Social baseline theory: The role of social proximity in emotion and economy of action. Soc. Pers. Psychol. Compass.

[58] McCall C (2017). Mapping social interactions: The science of proxemics. Curr. Top. Behav. Neurosci..

[59] Maister L, Tsakiris M (2016). Intimate imitation: Automatic motor imitation in romantic relationships. Cognition.

[60] Pellencin E, Paladino MP, Herbelin B, Serino A (2017). Social perception of others shapes one’s own multisensory peripersonal space. Cortex.

[61] Hayduk LA (1983). Personal space: Where we now stand. Psychol. Bull..

[62] Holt DJ (2014). Neural correlates of personal space intrusion. J. Neurosci..

[63] Vieira JB, Pierzchajlo SR, Mitchell DGV (2019). Neural correlates of social and non-social personal space intrusions: Role of defensive and peripersonal space systems in interpersonal distance regulation. Soc. Neurosci..

[64] Grivaz P, Blanke O, Serino A (2017). Common and distinct brain regions processing multisensory bodily signals for peripersonal space and body ownership. Neuroimage.

[65] Ferri F, Tajadura-Jiménez A, Väljamäe A, Vastano R, Costantini M (2015). Emotion-inducing approaching sounds shape the boundaries of multisensory peripersonal space. Neuropsychologia.

[66] Liebenthal E, Silbersweig D, Stern E (2016). The language, tone and prosody of emotions: neural substrates and dynamics of spoken-word emotion perception. Front. Neurosci..

[67] Engelen T, Zhan M, Sack AT, de Gelder B (2018). Dynamic interactions between emotion perception and action preparation for reacting to social threat: A Combined cTBS-fMRI study. eNeuro.

[68] Kennedy DP, Gläscher J, Tyszka JM, Adolphs R (2009). Personal space regulation by the human amygdala. Nat. Neurosci..

[69] Coker-Appiah D (2013). Looming animate and inanimate threats: The response of the amygdala and periaqueductal gray. Soc. Neurosci..

[70] Rymarczyk K, Żurawski Ł, Jankowiak-Siuda K, Szatkowska I (2019). Empathy in facial mimicry of fear and disgust: Simultaneous EMG-fMRI recordings during observation of static and dynamic facial expressions. Front. Psychol..

[71] Kim MJ (2014). Botulinum toxin-induced facial muscle paralysis affects amygdala responses to the perception of emotional expressions: Preliminary findings from an A-B-A design. Biol. Mood Anxiety Disord..

[72] Hennenlotter A (2009). The link between facial feedback and neural activity within central circuitries of emotion: New insights from botulinum toxin-induced denervation of frown muscles. Cereb. Cortex.

[73] Kappas A, Krämer NC (2011). Face-to-Face Communication over the Internet: Emotions in a Web of Culture, Language, and Technology.

[74] Faul F, Erdfelder E, Lang A-G, Buchner A (2007). G*Power 3: A flexible statistical power analysis program for the social, behavioral, and biomedical sciences. Behav. Res. Methods.

[75] Fridlund AJ, Cacioppo JT (1986). Guidelines for human electromyographic research. Psychophysiology.

[76] Brainard DH (1997). The psychophysics toolbox. Spat. Vis..

[77] Lundqvist D, Flykt A, Öhman A (1998). The Karolinska Directed Emotional Faces—KDEF.

[78] Kret ME, Sjak-Shie EE (2018). Preprocessing pupil size data: Guidelines and code. Behav. Res..

[79] Maris E, Oostenveld R (2007). Nonparametric statistical testing of EEG- and MEG-data. J. Neurosci. Methods.

[80] Vinding MC (2019). Attenuated beta rebound to proprioceptive afferent feedback in Parkinson’s disease. Sci. Rep..

