# Author Correction: Simulated proximity enhances perceptual and physiological responses to emotional facial expressions

**DOI:** 10.1038/s41598-022-08408-5

**Published:** 2022-03-17

**Authors:** Olena V. Bogdanova, Volodymyr B. Bogdanov, Luke E. Miller, Fadila Hadj‑Bouziane

**Affiliations:** 1 IMPACT Team, Lyon Neuroscience Research Center, INSERM, U1028, CNRS, UMR5292, University of Lyon, Bron Cedex, France; 2 Université de Bordeaux, Collège Science de la Sante, Institut Universitaire des Sciences de la Réadaptation, Handicap Activité Cognition Santé EA 4136, Bordeaux, France; 3 Donders Centre for Cognition of Radboud University in Nijmegen, Nijmegen, The Netherlands; 4 INCIA, CNRS UMR 5287, Université de Bordeaux, Bordeaux, France

Correction to: *Scientific Reports* 10.1038/s41598-021-03587-z, published online 07 January 2022

The original version of this Article contained errors in Figure 1, where panel (**B**) was a duplication of panel (**A**). The original Figure 1 and accompanying legend appear below.

**Figure 1:**
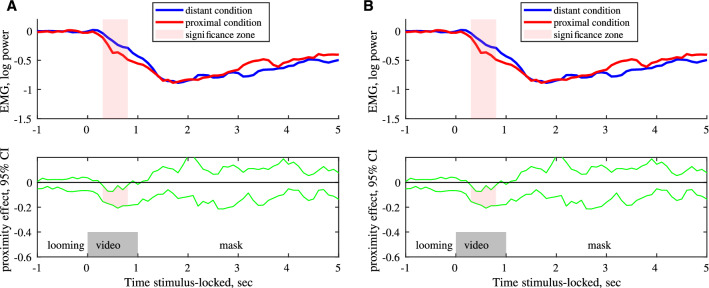
*Corrugator supercillii* activity during observation (**A**, n = 41) and imitation (**B**, n = 36) of happy faces. Upper row: the median of EMG activity for distant and proximal conditions. Lower row*:* 95% confidence intervals for effect of condition (proximal vs distant conditions). *Pale pink shadow* indicates clusters of data points with significant differences between proximal and distant conditions (A - p < 0.001 and B - p < 0.05 cluster level based on median bootstrap permutation test, n = 1000).

The original Article has been corrected.

