# Supplementary material for: Simulated proximity enhances perceptual and physiological responses to emotional facial expressions
Source: Sci Rep. 2022 Jan 7;12:109. doi: 10.1038/s41598-021-03587-z (PMC8741866; doi:10.1038/s41598-021-03587-z)
Supplement: Supplementary file 1 — Supplementary Information. [file 41598_2021_3587_MOESM1_ESM.docx]

Supplementary materials for the paper entitled

“SIMULATED PROXIMITY ENHANCES PERCEPTUAL AND PHYSIOLOGICAL RESPONSES TO EMOTIONAL FACIAL EXPRESSIONS”

Olena V. BOGDANOVA, Volodymyr B. BOGDANOV, Luke E. MILLER, Fadila HADJ-BOUZIANE

**Supplementary Figure 1.** C*orrugator supercillii* activity in 5 subjects, displaying an EMG activation during the imitation of happy faces.

**Upper row:** the median of EMG activity for distant and proximal conditions.

***Lower row:*** 95% confidence intervals for effect of condition (proximal vs distant conditions).


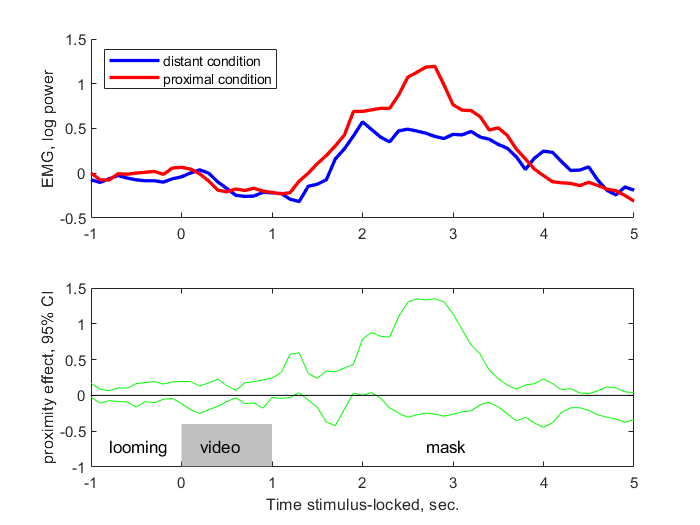


| **Supplementary figure 2.** 95% confidence intervals for proximity-independent c*orrugator supercillii* activity responses on emotional expressions presentation (angry, A,B, n=42 and happy C,D, n=36) during observation (A,C) or imitation (B,D) tasks: | |
| --- | --- |
| A  B | 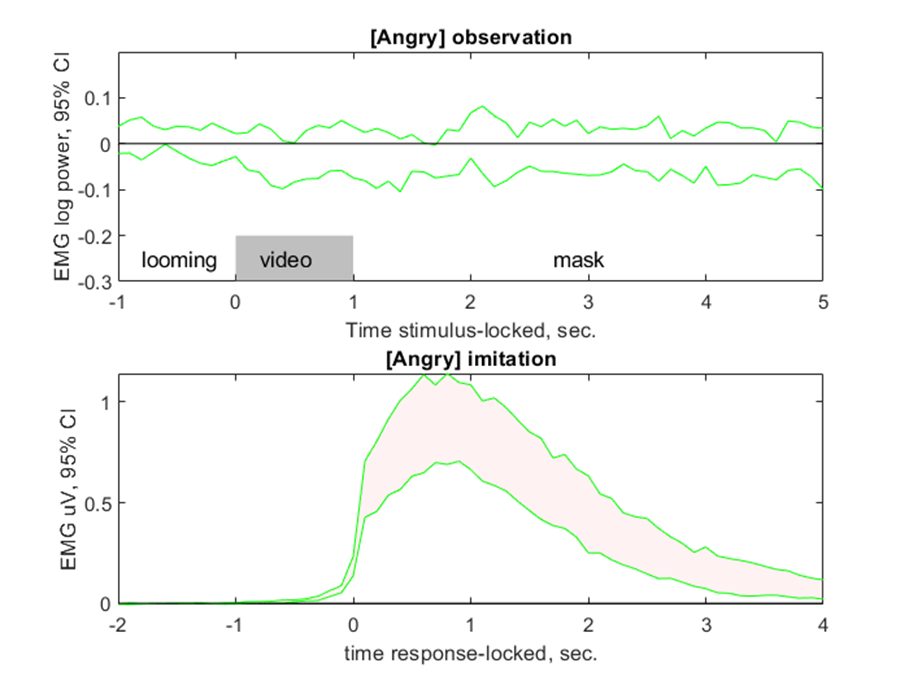 |
| C  D | 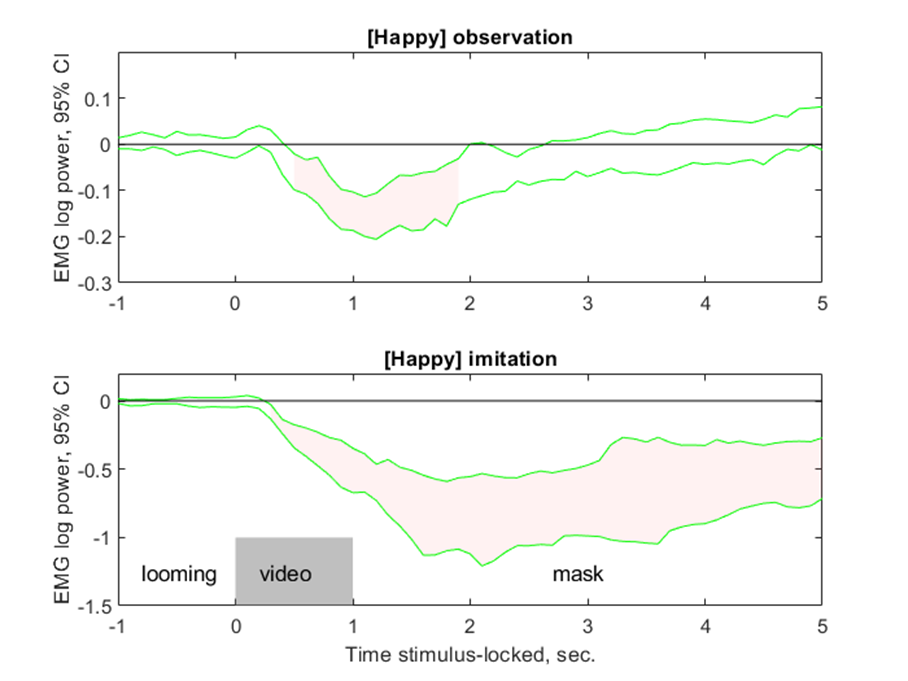 |

**Supplementary figure 3.** 95% confidence intervals for proximity-independent responses of pupil diameter on emotional stimuli presentation (n=41):

**upper row** effect of emotion (angry vs happy)

**lower row** effect of effect of session (imitation vs observation)


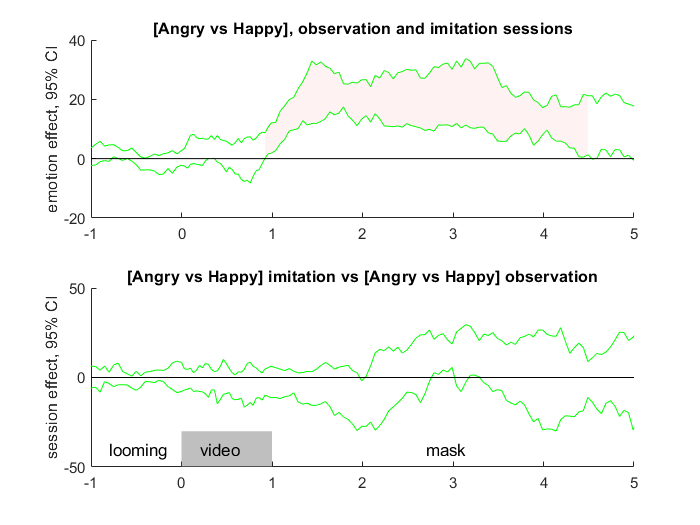


| **Supplementary figure 4.** No significant differences between the proximity effects on face muscular activity (A,B) and pupil diameter (C) responses recorded in proximity-enhancement, PE and no-enhancement, nE sub-category, respectively PE vs nE, trials | |
| --- | --- |
| 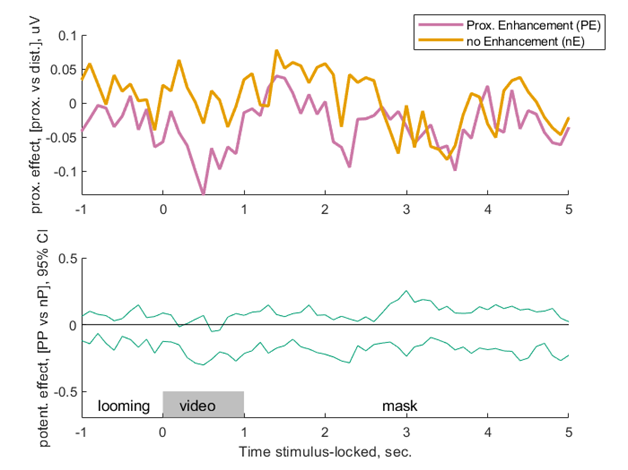 | 1. Proximity effect (difference between proximal and distant conditions) on corrugator supercillii activity during observation of happy faces (n=36):   The medians for PE and nE trials (upper row) and theirs 95% confidence intervals (lower row) |
| 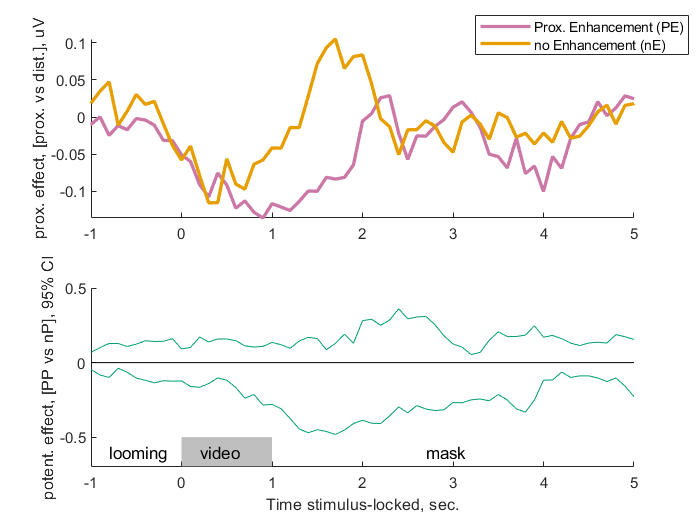 | 1. Proximity effect (difference between proximal and distant conditions) on corrugator supercillii activity during imitation of happy faces (n=36):   The medians for PE and nE trials (upper row) and theirs 95% confidence intervals (lower row) |
| 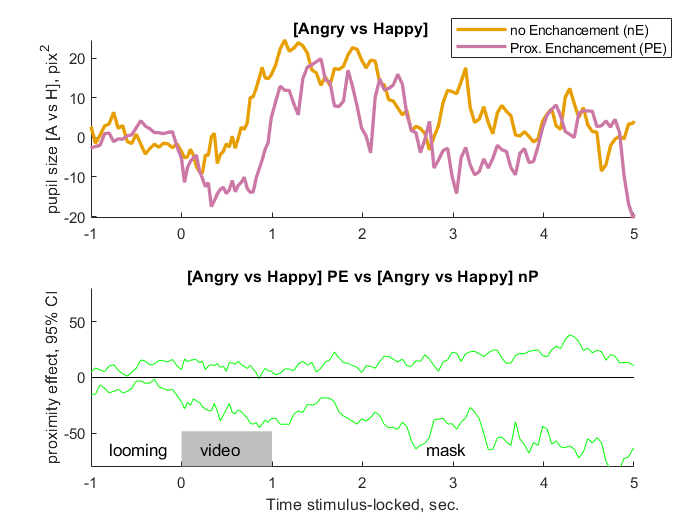 | 1. Proximity effect (difference between proximal and distant conditions) on pupil differential response pooled for imitation and observation sessions (n=41):   The medians for PE and nE trials (upper row) and theirs 95% confidence intervals (lower row) |
